# Supplementary material for: Reduction in social learning and increased policy uncertainty about harmful intent is associated with pre-existing paranoid beliefs: Evidence from modelling a modified serial dictator game
Source: PLoS Comput Biol. 2020 Oct 15;16(10):e1008372. doi: 10.1371/journal.pcbi.1008372 (PMC7591074; doi:10.1371/journal.pcbi.1008372)
Supplement: S1 Text — (DOCX) [file pcbi.1008372.s001.docx]

**S1 Text Task Instructions**

**Task A**

You will now take part in a series of trials with three players.

You will be playing with them one by one.

Each partner will play with you for 6 trials.

Their IDs have been hidden to preserve their anonymity.

Each trial has the same starting conditions:

You have been allocated a bonus of £**0.10**.

Your partner was allocated a bonus of £0.00.

In each trial, players could choose one of the following options:

Take £0.05 of your bonus and leave £0.05 for you

Take £0.10 of your bonus and leave £0.00 for you

Each of the players have already made their decisions.

At the end of the trials your bonuses will be added up and given to you.
