# Supplementary figures and images for: Reduction in social learning and increased policy uncertainty about harmful intent is associated with pre-existing paranoid beliefs: Evidence from modelling a modified serial dictator game
Source: PLoS Comput Biol. 2020 Oct 15;16(10):e1008372. doi: 10.1371/journal.pcbi.1008372 (PMC7591074; doi:10.1371/journal.pcbi.1008372)

**S2 Figure Task Schematic**

**
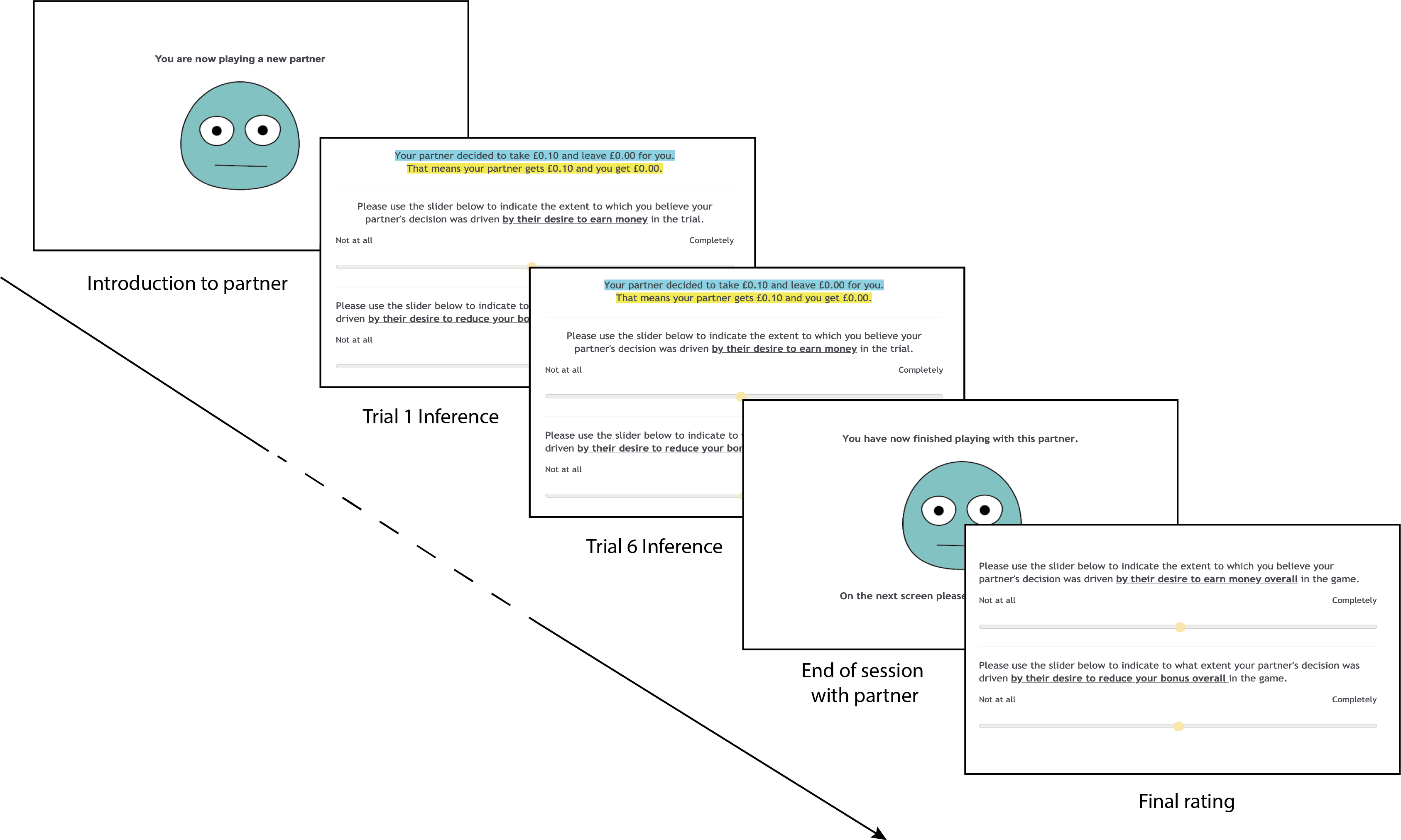
**

Supplement: S1 Fig — (DOCX) [file pcbi.1008372.s002.docx]

**S3 Figure Network metrics.**


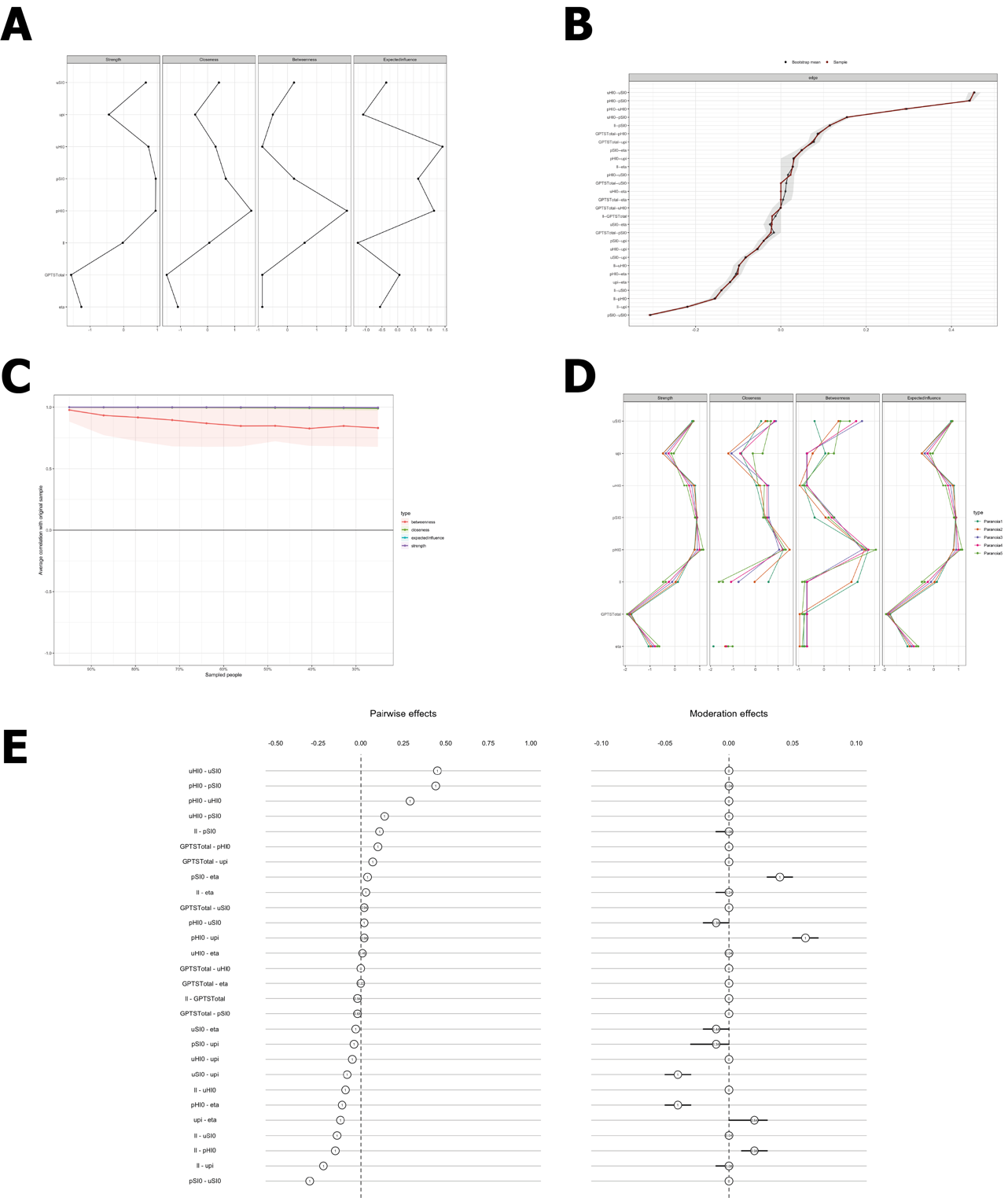

Supplement: S2 Fig — (A) Node centrality–strength, closeness, betweenness, and expected influence–of the overall model (Fig 3A). (B) Accuracy of the network when bootstrapped with 1000 sampling-with-replacement. Grey shaded area represented confidence intervals. (C) Stability of the strength, closeness, and betweenness of nodes in the network. (D) Node centrality by each model moderated at different degrees of prior paranoia. (DOCX) [file pcbi.1008372.s003.docx]

**S6 Figure Cluster densities between initial attribution probabilities and other parameters.**


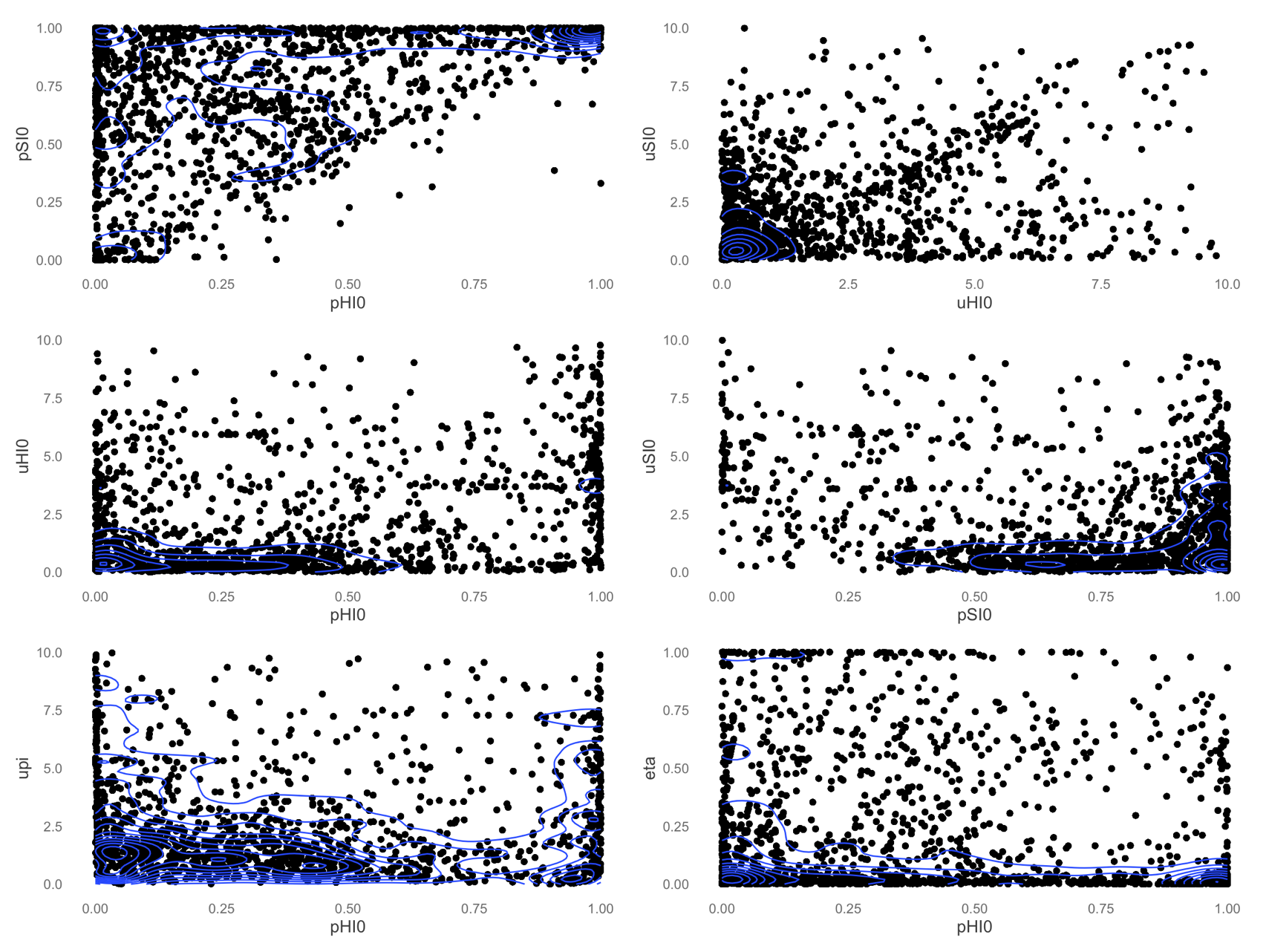

Supplement: S5 Fig — (DOCX) [file pcbi.1008372.s006.docx]

**S8 Figure Spearman correlations between GPTS score and latent parameters.**


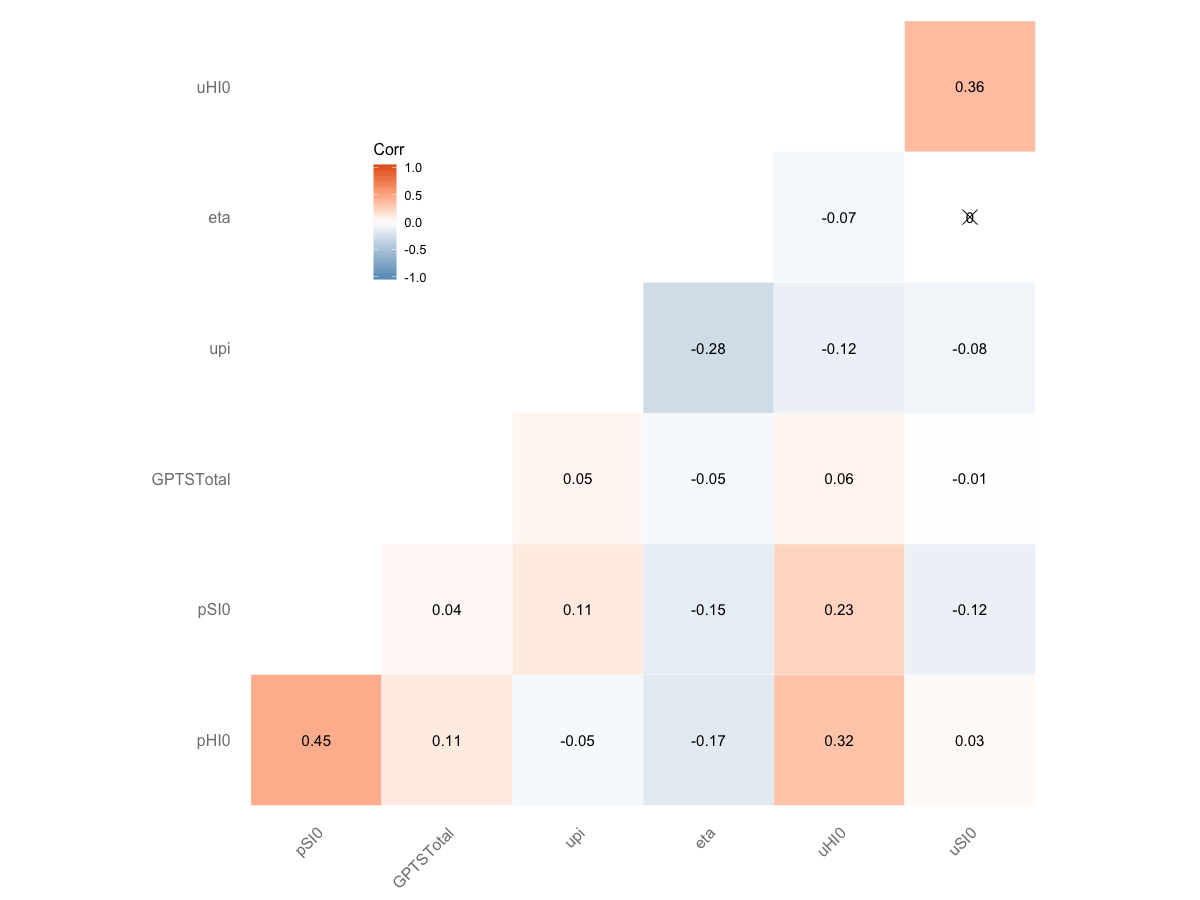

Supplement: S7 Fig — All other values are significant at least at the p<0.05 level. (DOCX) [file pcbi.1008372.s008.docx]
