# Supplementary material for: Reduction in social learning and increased policy uncertainty about harmful intent is associated with pre-existing paranoid beliefs: Evidence from modelling a modified serial dictator game
Source: PLoS Comput Biol. 2020 Oct 15;16(10):e1008372. doi: 10.1371/journal.pcbi.1008372 (PMC7591074; doi:10.1371/journal.pcbi.1008372)

**S4 Figure Correlations between simulated and real parameters (A) and bootstrapped estimates of each corresponding network (B).**


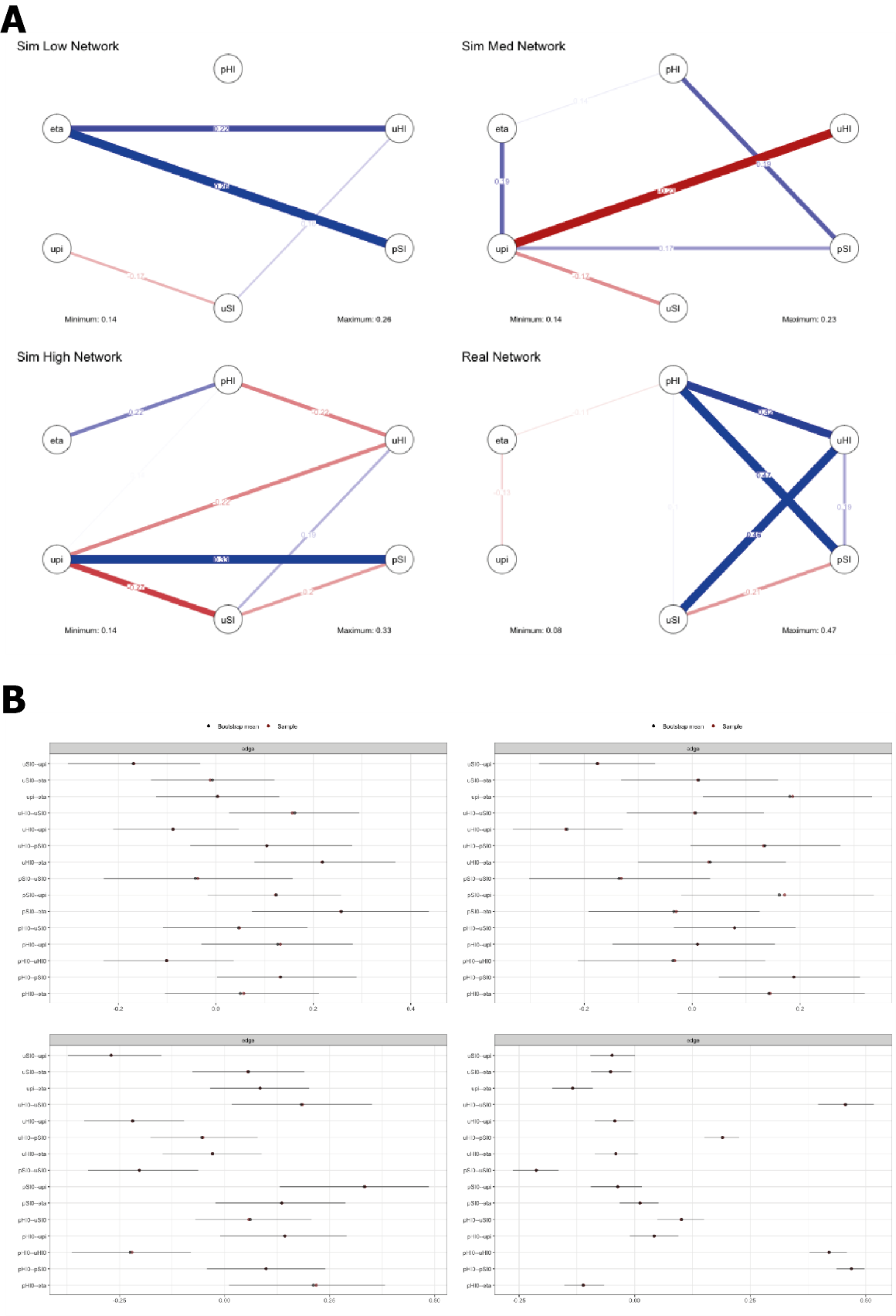

Supplement: S3 Fig — Low, Med, and High represent the clusters of pHI and pSI parameters used to simulate the data based on the differential clustering found in Fig 7. Each bootstrapped network analysis used 1000 bootstraps. (DOCX) [file pcbi.1008372.s004.docx]
