# Supplementary material for: Reduction in social learning and increased policy uncertainty about harmful intent is associated with pre-existing paranoid beliefs: Evidence from modelling a modified serial dictator game
Source: PLoS Comput Biol. 2020 Oct 15;16(10):e1008372. doi: 10.1371/journal.pcbi.1008372 (PMC7591074; doi:10.1371/journal.pcbi.1008372)
Supplement: S4 Fig — (DOCX) [file pcbi.1008372.s005.docx]

**S5 Figure Reduced Moderated Network Models with two (A & B), three (C), four (D), and five (E) variables included.**

**
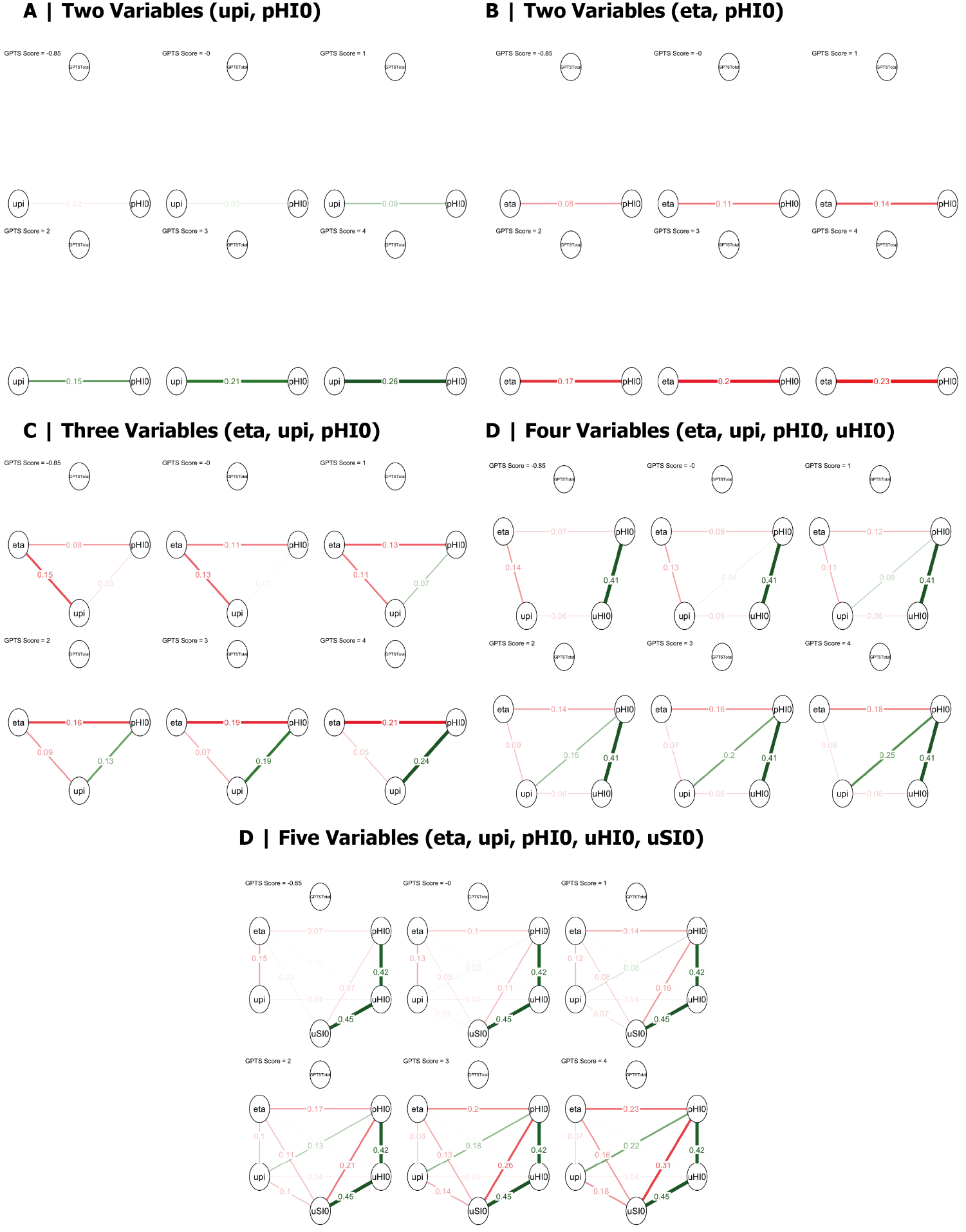
**
