# Supplementary material for: Reduction in social learning and increased policy uncertainty about harmful intent is associated with pre-existing paranoid beliefs: Evidence from modelling a modified serial dictator game
Source: PLoS Comput Biol. 2020 Oct 15;16(10):e1008372. doi: 10.1371/journal.pcbi.1008372 (PMC7591074; doi:10.1371/journal.pcbi.1008372)

**S7 Figure Spearman correlations between simulated participants and real participant attributions of harmful intent (red) and self-interest (blue) for each level of GPTS division as defined in Barnby et al., 2020.**


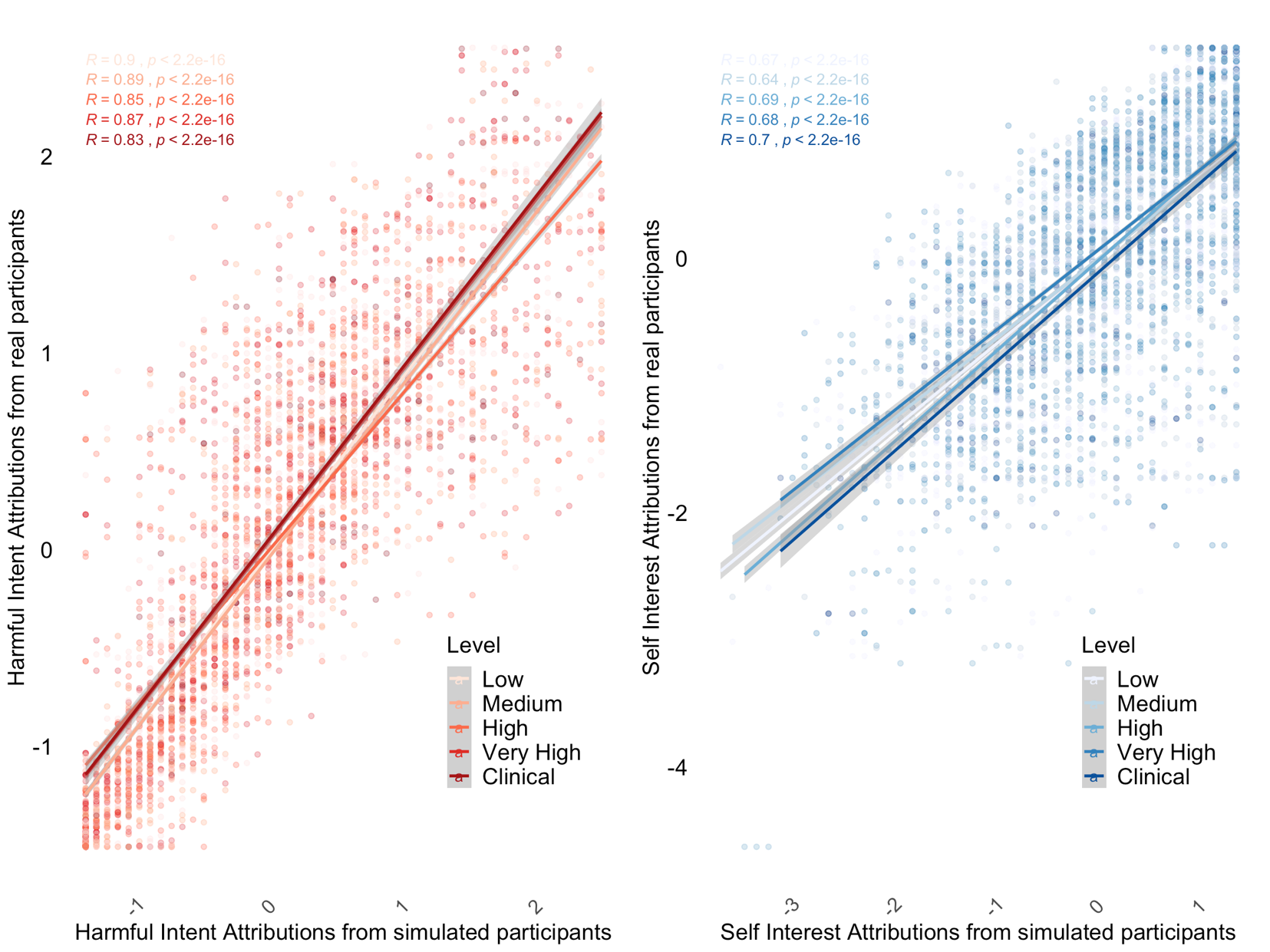

Supplement: S6 Fig — (DOCX) [file pcbi.1008372.s007.docx]
