# Supplementary material for: Reduction in social learning and increased policy uncertainty about harmful intent is associated with pre-existing paranoid beliefs: Evidence from modelling a modified serial dictator game
Source: PLoS Comput Biol. 2020 Oct 15;16(10):e1008372. doi: 10.1371/journal.pcbi.1008372 (PMC7591074; doi:10.1371/journal.pcbi.1008372)

**S9 Figure Spearman correlations between GPTS score, its component subscales and latent parameters.**


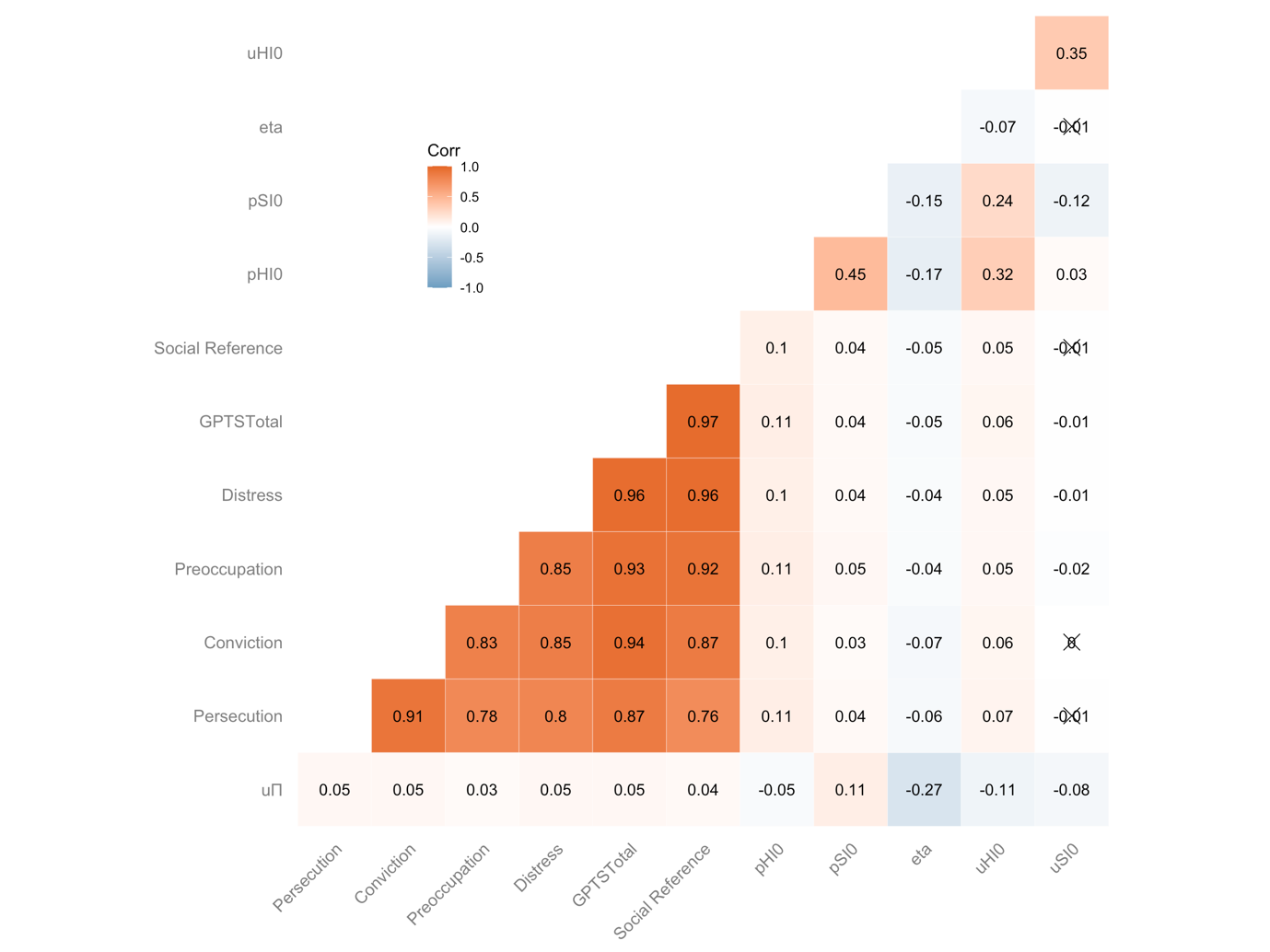

Supplement: S8 Fig — All other values are significant at least at the p<0.05 level. (DOCX) [file pcbi.1008372.s009.docx]
