# Supplementary material for: Reduction in social learning and increased policy uncertainty about harmful intent is associated with pre-existing paranoid beliefs: Evidence from modelling a modified serial dictator game
Source: PLoS Comput Biol. 2020 Oct 15;16(10):e1008372. doi: 10.1371/journal.pcbi.1008372 (PMC7591074; doi:10.1371/journal.pcbi.1008372)
Supplement: Attachment — Submitted filename: PLOSCompBiol_16062020_Paranoia_DictatorGame.pdf [file pcbi.1008372.s010.pdf]

REVIEW

"Reduction in social learning and policy uncertainty about intentional social threat underlies paranoia: evidence from modelling a modified serial dictator game."

RECOMMENDATION:

Minor Revision

OVERALL IMPRESSION

I would like to thank the authors for an exciting read! The paper was well structured and the methods were interesting and appropriate for the data they were used on. The points I am raising mainly concern clarity on a few details and open questions.

ISSUES RAISED BY REVIEWER

1. ***Introduction and Methods:***
    1) Could you provide the reader with the instructions the participants received regarding the game and the dictators? This is not only important for replication of these results but also in order to understand better, what the participants might have based their inferences on.
    2) Did participants know that they would be playing against different types of dictators?
    3) Do you have any demographic data that you can provide for this sample?
    4) Why did you only use the two types of attributions you mentioned? Couldn't some participants have assumed that there were no real intentions behind the dictator behavior but that it was just how the game was set up?
    5) You treat HI and SI as independent could you elaborate on why you assumed that these two types of attributions are independent? After all they did correlate with each-other and also conceptually they do seem to share common ground.

2. ***Introduction, Lines 38-43:*** *"More recently, Barnby et al. (2020) extended this work and additionally found that individuals high in paranoia were more likely to specifically reduce high harmful intent attributions after an initial peak when interacting with partners who were consistently fair, when compared to people low in paranoia, potentially suggesting increased volatility of paranoid social inferences."*
I have read this sentence three times now but still cannot follow the reasoning. Maybe it's too long of a sentence. Would you mind making it easier to follow what you found and how this relates to your hypothesis regarding volatility?

3. ***Introduction, Lines 89-90:*** "*Second, we tested whether baseline harmful intent attributions about the partner depended on pre-existing paranoia."*
What do you mean by baseline? The very first attribution?

4. ***Table 1, uHI_0:*** "*Greater uHI0 denotes weaker assumptions harmful intent, and hence greater willingness to believe that a Dictator less generous than expected has higher intent to harm."*
Shouldn't this be the other way around? Intuitively, a larger spread of the distribution should be linked to greater uncertainty regarding the harmful intent attribution, which should result in a smaller likelihood of this attribution being made?

5. ***Table 1, uΠ (Partner policy uncertainty):***
Here you are not describing what type of quantity this is. Uncertainty has been analogous to the spread of a distribution before, which is not the case here. For clarity and consistency, it might be beneficial to specify this here.

6. ***Table 1, η: "A higher η leads the starting assumptions of dictators after the first one seen to be influenced by the evidence seen. It can be thought of as a strength of belief that the Dictators seen during the experiment will resemble each other."***

These two sentences sounded contradictory to me at first and second glance. Maybe because you don't state what you mean by "evidence". My first interpretation was that you mean trial-by-trial information but you might mean something different?

7. ***Results, general:***
   1) Would you mind stating effect-sized for the results you are reporting?
   2) I know from your previous paper that you divided participants into "low", "medium", "high", "very high", "clinical" regarding GPTS scores. However, this is not clear at all when only reading this paper. Could you mention that somewhere, i.e. what the score cut-offs for each of these groups were and why you chose these particular cut-offs and bins?
   3) How many participants attributed HI and SI equally often or seemed to choose their attributions randomly? It might be possible that some participants might have believed that this was all computer-generated and thus did not really try to answer these attribution questions. Would that be reflected in the policies and if so, could you provide a plot for the mean partner policies over trials?

8. ***Results, Lines 111-116:***

"Linear mixed model analyses using ID…"

1) could you spell out "ID" the first time you use it in the text body? I assume you mean the participant identification number, correct?

"…as a random term suggest that as trials progressed overall from one to eighteen (-0.012, 95%CI: -0.021, -0.003) and at higher values of the GPTS (-0.005, 95%CI: -0.007, -0.003) participants were less able to be predicted by our model, …"

2) Is this based on a regression on the GPTS scores or based on divisions of the GPTS scores? If the latter is the case, how did it enter the mixed model?
3) Across all types of dictators?

"…whereas our model was better able to predict behaviour from partially fair (0.43, 95%CI: 0.32, 0.55) and unfair (0.31, 95%CI: 0.25, 0.55) dictators than fair dictators."

4) Better than what? And in this case now across all GPTS scores?

9. ***Figure 1, general:***

It would increase clarity greatly if you could add titles to each subfigure and in the legend mention that the horizontal line refers to chance-level.

10. ***Figure 1, C and D:***

I assume that the Scale (Low to Clinical) relates to the GPTS scores? Is that the way you divided the groups then? This does not immediately become clear when looking at this figure. In the legend you refer to plotting the mean log likelihood for each dictator, I cannot see this here and also cannot see anything with reference to this scale.

11. ***Results, , Lines 138-144:*** *"Simulated (n = 1754) harmful intent attributions and self-interest attributions were only slightly negatively correlated with each other overall (rho = -0.06, p < 0.001)."*

1) This implies that you expected and were aiming for this negative correlation. Is that so and why?

"Pre-existing paranoia in all dictator conditions increased harmful intent attributions (Unfair: 0.21, 95%CI: 0.18, 0.25; Partially Fair: 0.19, 95%CI: 0.16, 0.23; Fair: 0.18, 95%CI: 0.15, 0.22). "

2) Paranoia increasing harmful intent attributions implies that they were lower at some point before where there was no paranoia, because the word is used as a verb. It's also not clear in reference to what the attributions were increased, are you comparing the clinical GPTS scores to all others or is this across all scores? Does this result refer to all or only a specific dictator?

"Pre-existing paranoia was only a predictor of slightly reduced self-interest attributions in unfair dictators only (-0.08, 95%CI: -0.12, -0.04), using individual cumulative link models with age and first dictator exposure as additional predictors."

3) I apologize for my ignorance, but I could not find out what individual cumulative link models are, would you mind explaining that in one or two sentences or provide a reference?

12. ***Results, Lines 437-440:*** *"The prior belief over both HI and SI can then be written as a product of the independent prior probabilities, p(0)HI * p(0)SI . This assumption of independence is conservative, minimizing the number of free parameters.".*
and ***Lines 440-448:*** "*We then assumed that HI=0, SI=0 resulted would correspond to a preference in giving r=n (of n, i.e. 100%) to the participant ('self-sacrifice'), HI=1, SI=1 to a high preference for giving r=1 (of n, i.e. 0%) to the participant, but very high SI or HI with moderate scores on the other dimension we sufficient for a substantial probability of r=1 of n (i.e. 0%).".*

1) Maybe I am misunderstanding something here, but first you state that you treat HI and SI as independent but in the next section you state that their mapping from dictator behavior to beliefs are the same. Are you not then saying that they actually are NOT in fact independent?
2) I assume "r" stands for reward. This is the first time this comes up in the text body, please indicate its meaning when you are using it for the first time.

13. ***Results, Lines 458-460:*** "*This means that every modelled participant had the same basic repertoire of attribute behaviour available to them, and that HI and SI can be seen as ideographically scaled."*
Apologies for my ignorance again. What does ideologically scaled mean?

14. ***Results, Lines 496-498:* "***In contrast to a model selection approach, the ability of a model to simulate data is necessary to assess its validity and falsification (Palminteri et al., 2017)."*
Why is that 'in contrast to model selection?' Are they competing approaches?

15. ***Results, Lines 279-282:*** *"Our findings converge with the idea that paranoia strengthens higher-level beliefs about others to influence momentary inferences regardless of a partner's behaviour (Wellstein et al., 2019)."*
Based on what results do you reach this conclusion? It is not entirely clear to me.
