# Supplementary material for: Reduction in social learning and increased policy uncertainty about harmful intent is associated with pre-existing paranoid beliefs: Evidence from modelling a modified serial dictator game
Source: PLoS Comput Biol. 2020 Oct 15;16(10):e1008372. doi: 10.1371/journal.pcbi.1008372 (PMC7591074; doi:10.1371/journal.pcbi.1008372)
Supplement: Attachment — Submitted filename: ReviewerResponses_PLOSCB.docx [file pcbi.1008372.s011.docx]

**Editor Comments**

I have an additional comment, regarding the estimation of network interactions. You can have mediated or directed interactions, and for this you normally use partial correlations. On the other hand you can have associations which go beyond pairwise interactions (regardless of the fact that they are mediated or not). This is the case when you have latent variables which jointly influence a subset of variables in the system, but you can also have higher order effects within the variables in the system, for example two variables sharing joint information on a third one. These types of interactions confound the estimation of pairwise interactions, also when conditioning for the action of other single variables. See for example

Hoffmann, T., Peel, L., Lambiotte, R., & Jones, N. S. (2020). Community detection in networks without observing edges. Science Advances, 6(4), eaav1478. doi:10.1126/sciadv.aav1478

Stramaglia, S., Cortes, J. M., & Marinazzo, D. (2014). Synergy and redundancy in the Granger causal analysis of dynamical networks. *New Journal of Physics*, *16*(10), 105003. <http://dx.doi.org/10.1088/1367-2630/16/10/105003>

Do you control for this type of interactions? Do you expect them?

We thank the editor for these observations, and the relevant references. In order to guard against artefacts of the partial correlation analyses, avoid collider bias, and control for redundancy in networks we proceeded as follows.

We first address collider bias. The Psychonetrics package (Epskamp et al., 2020), Bootnet package (Epskamp et al., 2018), and Moderated Network Model package (Haslbeck et al., 2019) all generate networks of partial correlations which calculate pairwise relationships between variables while controlling for all other pairwise relationships in the network. Therefore, each edge observed is a partial correlation corrected for other pairwise correlations. However, nonetheless, networks dynamics still open the possibility of collider bias in regard to two strong correlations between two variables when regressed on a common variable (e.g. a positive correlation between A and C, and B and C, may generate a spurious negative correlation between A and B). This is visualised in the network as a strong opposite correlation to the direction of the two other strong correlations. However, we do not see this in any of our networks.

In order to investigate these issues further, we included a Spearman’s correlation matrix in the supplementary materials. Reassuringly, we see that GPTS score (and its constituent subscales) has associations with parameters in the same direction as per our original analysis and our conclusions. Specifically, GPTS score has a significant negative association with learning, and a significant positive association with partner policy uncertainty, harmful intent attributions, uncertainty over harmful intent attributions.

We next address artefactual relationships. We also had the same concerns as reviewer 3 against spurious correlations induced by the modelling and model-fitting process. We checked for this in our original submission by running simulations of the network using high density clusters of our latent parameter estimates (see supplementary materials). Consistent relationships between variables that appear due to the refitting process, that are the same in direction as with the network using real data, would be a cause for concern and might suggest artefactual relationships in the fitting process. However, we do not see this consistently across the networks.

Finally, to overcome the issue of redundancy of variables listed in Stramaglia et al. (2014), we have included networks with less parameters in the supplementary materials. We have included these to demonstrate that, in the absence of additional parameters that might confound the result, the relationships in the network moderated by GPTS score that are potentially less reliable (pHI0 -> eta, pHI0 -> upi) are consistent when removing parameters. We have referenced this in the main text (line 293-301):

“In addition, it may be that the complexity of the network itself is generating moderated effects between parameters due to redundant inclusion of variables around the relationships of interest (Stramaglia et al., 2014). To control for this possibility, we ran the Moderated Network Model with two, three, four, and five variables in the network to check whether the relationships between learning rate (eta), partner policy uncertainty (upi), and probability of attributing harmful intent (pHI0) still existed in absence of other relationships (see Supplementary Materials). We found that the variables in question were retained and were moderated in the same direction by GPTS score in all network models.”

**Reviewer #1**

The authors of “Reduction in social learning and policy uncertainty about intentional social threat underlies paranoia: evidence from modelling a modified serial dictator game” present a computational model to explain how pre-existing paranoia impacts social learning and perception of others’ behaviour.

This is a well written paper and tackles an important aspect of paranoia, namely one’s uncertainty about the others’ action policies. I appreciate the inclusion of in-silico data to reproduce the major behavioural trends observed empirically. I have a few questions related to the study design and the predictive validity of the model parameters.

1. Paranoia using the Green Paranoid Thoughts Scale (GPTS): First, different aspects of pre-existing paranoia might determine orthogonal aspects of social learning and decision-making (hence the question below related to subgroups). For example, an increased frequency in paranoid thoughts may relate to more uncertain beliefs about others’ intentions while increased conviction of paranoid thoughts may be predicted by overly high precision of predictions and therefore reduced learning rates and belief flexibility. Thus, my question is, does the GPTS scale you have used differentiate between these aspects of paranoia? And if so, what aspect of paranoia is predicted by which parameter of your model?

We thank the reviewer for this interesting hypothesis – something that the authors also share. The Green Paranoid Thoughts Scale is comprised of social reference and persecutory ideation subscales, although the scale can also be divided into preoccupation, distress, and conviction subscales (Green et al., 2008). It is the most reliable and valid self-report measure for paranoia as a whole (Statham et al., 2019), although has been more recently revised (Freeman et al., 2020) after we had already begun putting together our prior experiment (Barnby et al., 2020) and this present study.

In this model, and in our previous paper (Barnby et al., 2020), we use the GPTS as a whole scale which has shown reliable associated with harmful intent attributions, but not self-interest attributions as predicted. Future work that we’re conducting aims to try and decipher how persecutory ideation alone influences live social cognition.

We have gone back to the individual items on the GPTS and scored them into their component parts and now included a spearman correlation matrix of all parameters and subscale associations (Appendix F). We have made reference to this in the text (line 251).

“In addition, we examined parameters in relation to the GPTS subscales, specifically its two main components, Social Reference and Persecutory Ideation, and when dividing the GPTS into scales of Conviction, Preoccupation, and Distress. When running spearman correlations, all subscales were highly correlated with each other (rho = 0.76 – 0.97, ps < 0.001), and all subscales were associated with the parameters to the same magnitude and direction as the GPTS total score (see Supplementary Materials, Appendix F).”

As with the GPTS total score, all subscales correlate with parameters to the same magnitude and in the same direction. While it was not the aim of the current study, we hope that future experiments are able to better tease apart these aspects of paranoia.

2. Second, was the assessment of paranoia performed at multiple time points? In some cases, a brief expression of paranoia may reflect a period of high stress and may be associated with an increased perception of volatility (and thus uncertainty about others) while a stable and prolonged expression of paranoia may reflect overly precise higher-level beliefs and reduced learning rates. Can you differentiate between state and trait?

The attribution of harmful intent measured via the serial Dictator game is in itself a state paranoid attribution – it is an inference that a behaviour was intentionally harmful. Paranoia was measured as both a trait (Green Paranoid Thoughts Scale; Green et al., 2008) and state (serial Dictator Game; Barnby et al., 2020) in two independent samples. The GPTS was measured seven days prior to the state measure of paranoia in each sample – one sample of around 900 people were recorded in September 2018, and the second sample of around 900 new people tested in April 2018. Unfortunately, we cannot control for the fact that the GPTS may be detecting periods of elevated stress and paranoid beliefs in some of the population dependent on their circumstances, but the GPTS phrases its constituent items to assess dispositions over the past month, not events that are occurring in that moment for a participant. The GPTS has shown to be the most reliable and valid self-assessment for the construct of paranoia (Statham et al., 2019).

We have made the task procedure clearer in the methods.

3. Experimental Paradigm: It is not clear from the description of the task whether this was a reciprocal exchange or rather used a fixed input structure. Related to this, it unclear whether deception was used. It would help to include the task instructions.

The participant had no say over the amount of money they received – in a classic Dictator game design - and were only required to infer their partners intentions which is a modification we introduced previously (Barnby et al., 2020). This used a fixed-input structure; participants always saw unfair, partially fair, and fair partners in a random order. After being in this role the participant was then required to make six dictator decision for a *new* partner following their role as the recipient. We informed participants that their decisions would be used with other participants in the game. This uses an ‘ex-post’ matching design which avoids the use of deception (cf. Horton, J. J., Rand, D. G. & Zeckhauser, R. J. The online laboratory: conducting experiments in a real labor market. *Exp. Econ.* **14**, 399–425 (2011)), and means that the answers participants received in the first part of the game were at some point made by other participants during all previous iterations of the game when choosing their decisions as dictator. We have made this clearer in the methods and described it further in lines 421-423:

“After making all 42 attributions (two trial attributions for each of the 6 trials over 3 partners, plus three additional overall attributions for each partner), participants were put in the role of the dictator for 6 trials – whether to make a fair or unfair split of £0.10. Participants were first asked to choose an avatar from nine different cartoon faces before deciding on their 6 different splits. These dictator decisions were not used for analysis but were collected in the first phase of the game to match subsequent participants with decisions from real partners using an ‘ex-post’ matching design.”

We have also included a task structure example and instructions given to the participants in the supplementary materials.

4. Can the computational model parameters identify different mechanisms of paranoia affecting social inference and decision-making? The effect sizes related to the predictive validity of the model parameters seems to be rather low. Do the authors have an interpretation for this? Given the current effect sizes, do you think the model can in theory capture individual differences in what aspect of social learning is impacted by paranoia?

The reviewer is right to point out that the effect sizes involved are rather low, and this is because of the limited reliability, at the individual level, of parameter estimates. The scale of the experiment meant that we could only administer 18 trials per participant. Therefore, again the reviewer is right that this format is not optimal for capturing individual differences, in the sense of reliably measuring where an individual is to be placed in the population distribution of parameters (and hence of individual learning and decision-making mechanisms associated with a particular, reliably measured Green scale score). Indeed, it is the presence of numerous individuals per quantile of paranoia scores which allows us to reliably comment on individual differences in the sense of the typical cognitive mechanisms associated with lower vs. higher paranoia scores. It is possible to posit a number of ‘common sense’ relationships a priori between paranoia and cognitive processes such as learning. Here, our modelling analysis selected amongst such alternatives; for example, it provided evidence that more suspicious individuals expect a larger variability in other’s actions; if observed actions convey less information about future actions, it would make sense not to revise too much beliefs about others between encounters, as shown in our analysis by the reduced learning rates in this situation.

5. Interpretability of network modelling results: These are a very useful representation of the interactions between parameters, but it would be helpful to filter out these interactions by which connections can be recovered through simulations. As you have pointed out, the interpretation of these results is confused by general correlations between the parameters of the model. These identifiability issues would be clarified using this simulations approach.

We use a simulations approach listed in the supplementary materials to address this issue as much as possible. We approximated the macroscopic distribution of parameters in the population by clustering generative parameters in a manner roughly similar to the real results, simulation and re-fitting data. Hence, we aimed to understand whether the network modelling and computational modelling process itself may produce the same correlations we observe, and therefore whether the modelling process itself is producing spurious correlations. The simulations produced very little evidence that the correlations we recovered were spurious, aside from the correlations mentioned in the text. We have included this reference to the supplementary materials in the text.

While we thank the reviewer for the suggestion to use parameters of a statistical structure that does not include higher-order interactions, generating data from these parameters, re-fitting the model, and seeing which network edges are recovered in the re-fitted parameters. However, we respectfully believe that this approach would not be convincing. The noise in experimental measurement (due to the small number of trials, etc.) almost certainly means that correlations are seriously degraded by the measurement-plus-fitting process. Therefore, recovering correlations is likely to require stronger ‘ground truth’ correlations than seen in our inferential statistics.

However, to provide additional reassurance, we have included in the supplementary materials the general correlation matrix between parameters and GPTS score (Appendix E), in addition to network models that have two, three, four, and five variables included in the network to establish whether the number of variables included in the network was influencing the moderated relationships of interest (Appendix G), however we found the moderation over these relationships was maintained even when other parameters were removed from the network.

I also have a few minor questions and comments:

1. What do you make of the consistent age effects observed?

In this present study we find that age only slightly predicted uHI0, uSI0 and learning. This may be reflective of overall reductions in risk of persecutory ideation as age increases observed in epidemiological studies (e.g. Freeman et al., 2016) – although these previous studies were mainly tracking trait paranoia, not experiences in the moment. Thus, it’s plausible to suggest that age may have an effect on paranoid ideation but may not interact with momentary paranoia to the same degree. This hypothesis requires further follow up work to understand how age might intersect with trait and state experiences, although we have some converging data from other studies to suggest this might be the case for other trait aspects - for example, anxiety and depression were highly related to trait paranoia in our previous study (Barnby et al., 2020) and reduced with age, but did not explain state, or live, paranoia in our task (Barnby et al., 2020).

2. I had the feeling that precision and uncertainty are sometimes used interchangeably, but I think it is important to be precise about what aspects of learning they refer to. For example, one may be overly confident about expecting harmful intent while still being uncertain about the dictator strategy at a given moment in time (i.e., potentially due to an enhanced perception of volatility). These two things may also be mutually exclusive and reflect different mechanisms of aberrant social learning linked to paranoia.

This is a good point, and it relates to the point of reviewer 3 that the description of parameters that we provide could be clearer. Indeed, we included three different uncertainty parameters precisely to differentiate between belief precision (or interchangeably its inverse, belief uncertainty) in these different domains. If one had high confidence about expecting harmful intent, one would have a low uHI0 parameter, i.e. low uncertainty of harmful intent beliefs. On the other hand, the same person could be very uncertain about what the dictator would do, captured in a high value of uΠ, the policy uncertainty. The hypothesis of the reviewer that these might be ‘mutually exclusive’ would then be reflected in an anticorrelation of uHI0 and uΠ in the population. As it happens, we did not find such an anticorrelation.

We have provided additional explanation in the Introduction as follows:

“We applied the model to estimate initial attributional strength (pHI0, pSI0) and inverse precision (uHI0, uSI0), in addition to how informative a partner’s behaviours were in regard to a change in intentions (uΠ) and how often attributions were updated from one dictator to the next (η). We thus used a spectrum of parameters describing uncertainty of different expectations. For example, a participant might be quite certain that others have high harmful intent (low uHI0) but be very uncertain about their partner’s actions (policy or strategy), reflected in a high value of uΠ. This detailed parameterization then allowed us to use the population variation of these parameters to infer whether a more limited range of cognitive styles in fact obtains, for example whether uncertainty into all types of beliefs vary together. The key parts of the model are described in Table 1 and full details of model development are given in Methods.

**Reviewer #2**

The paper describe a computational analysis of previously collected behavioral data in a study about paranoia that used a modified serial dictator game. Using a generative Bayesian model, those new results demonstrate how paranoia may be associated with uncertainty about other’s action, moderate the relationship between learning rates and harmful intent attributions, and how the intention attributions are related to their precision.

This is a timely piece combining computational psychiatry, social interactive task, and network analyses. The text is pleasant to read thanks to clear hypotheses and interpretations. It is also great to see both pre-registration and open source code. Since the manuscript is quite dense, it would just benefit from some clarification, especially in the method section. Overall, the article should meet the standard for publication in PLoS Computational Biology after some minor revisions.

Main points

- The causal claims like “underly” (in the title) or “predict” (in the text) may be tempered since there is no empirical demonstration about the underlying causal mechanisms of paranoïa.

We have adjusted the title and the text to remove ‘predict’ and ‘underly’ and replaced it with ‘associated with’.

- It is difficult to conclude about a “domain-general alteration” since there is no “non-social” context tested in the current study. To what extend paranoïa could also apply to harmful catastrophe (e.g. earthquakes)?

We agree with the reviewer and have adjusted all reference to domain-general alterations to make it clear we mean non-paranoid inferences about another. When we refer to domain-general alterations we mean in comparison to other studies that have looked at domain general changes as opposed to social changes. Paranoia is inherently a social fear about the inference of intentional harm placed upon a behaviour. Earthquakes themselves may make individuals anxious, but unless an individual felt the earthquake was caused by someone on purpose for the intended harm to an individual or group of individuals, this wouldn’t be classed as paranoia. We wanted to draw a different between specific social changes and previously non-social evidence, suggesting that is paranoia could be explained by general changes, then both of our attributional measures should have been manipulated in the same direction by pre-existing paranoia, although they were not, and infact moved in opposite directions. We suggest this is evidence of the differential importance of this social information in gauging interaction with a social other.

- The “Bayesian Brain” needs to be introduced more clearly. There is a common map-territory fallacy in the domain when papers apply Bayesian methods and simultaneously support a Bayesian perspective on what the brain does. The notion of “engram encoding probability distributions” should be especially more detailed.

We thank the reviewer for drawing attention to the need for clarification here. Indeed, in this study we used ‘Bayesian brain’ to refer to the former, which is, describing neurocognition as appropriate updating of uncertain beliefs on the basis of observations. Such appropriate updating is described by Bayes’ rule, and hence the ‘core engine’ of our model was indeed Bayes rule, as per Equation 4. Here we did not concern ourselves at all with the neural or algorithmic machinery behind belief updating such Eq. 4. We certainly do not claim that the brain performs the exact calculation of Eq. 4 – it may perform any number of approximations to such calculations, which are debated in the literature. We have now clarified, to avoid confusion with usage of Bayesian analytic methods:

‘Explanations for paranoia have increasingly focused on the role of learning and have been modelled based on the hypothesis that the brain processes uncertain information and appropriately revises beliefs depending on their baseline certainty and the impact of observations. This is the ‘Bayesian Brain hypothesis’, as appropriate revision of probabilistic beliefs is given by Bayes’ rule. In these models, predictions or ‘beliefs’ regarding the environment are generated and updated based on incoming prediction errors.’

We further explain, regarding engrams of probabilistic beliefs:

“Beliefs here are not necessarily, representational propositions but 'effective' beliefs, that is, engrams encoding probability distributions, necessary for performing approximate probabilistic information processing. How such engrams may be implemented in the brain is a matter of debate. Much evidence suggests that the brain encodes expectations about what will be observed (Friston & Stephan, 2007; Schultz et al., 1997) but it is less certain as to how the shape of belief distributions is represented. Some hold that the width of belief distributions is represented as activity explicitly reflecting precision, while others claim that the brain may estimate uncertainty ‘on the fly’ by sampling from available memories or other representations (Aitchinson & Lengyel, 2017; Echeveste et al., 2020). In our case, the nature of these engrams of expectation and uncertainty is irrelevant, but their function to provide weights for the updating of different beliefs is crucial.”

Minor points

- The sentence at lines 39-43 is hard to process.

This has been modified to make it easier to understand.

- The modification of the dictator game is not explicitly described, even in the methods (lines 383-393).

We have included the full description of the modified Dictator game in the methods (399-423), in addition to including participant instructions and a task schematic in the supplementary materials.

- Sometimes the term “Participants” is used in the simulation context and it must be clear when we are talking about “Virtual Participants” or “Real Participants” (e.g. Fig 2 line 169).

We have now clarified that simulated data refers to ‘virtual participants’ in the results.

- The use of acronyms make the piece somehow hard to follow. e.g. “GPTS” is defined only in the methods (line 368) and could easily be introduced in the main body of the text, e.g. line 88-94 with “pre-existing paranoid belief (as measured by the GPTS questionnaire)” or on those lines. This would drastically facilitate readers understanding. Same with parameters acronyms, which makes sense only after reading the methods. e.g. “pHI0” could be replace by “the initial probability of Harmful Intent (pHI0)” at first so that reader can get familiar with the notations.

Acronyms have been introduced earlier and fleshed out in the text to avoid confusion for the reader. We have also introduced acronyms in the results section when they first appear, and additionally in the table at the end of the introduction.

- Statistics
- Line 110: What can be concluded by the fact that log likelihood did not drop below -4.394? For people not familiar with Bayesian statistics, it may be nice to just briefly explain the motivation to report this.

We have made this clearer in the text and figure legends that -4.394 denotes the point at which the model would be accounting for the data by chance.

- Lines 111-115 / Lines 191-193: How can we interpret that as trials progress and GPTS increase, model fit is worse? To what extend the correlation between model fit and experimental factors is not problematic?

Much like Nour (et al., 2018), we might interpret the result between loglikelihood and GPTS score that participants played much less like a ‘Bayesian’ observer, as the model is built to represent. We might find this as evidence of convergence with previous studies, finding that pre-existing paranoid beliefs are less able to be fit to a Bayesian ideal. We will make a mention of this in the discussion (line 298 – 300)

- Lines 223-234: This paragraph should more clearly written, especially regarding the cluster definition and rational of the analysis.

We have made this paragraph clearer to make the rationale for our simulation analysis clearer.

- Difference between “pairwise” and “moderation” should be better explained.

In the figure legend of figure 5 I have made the pairwise and moderation definitions clearer.

- What are the grid search parameters for the MAP on (line 492)?

- Figure
- 1 & 2: X axis labels have missing spaces

We have increased the gradations for the x-axis labels to make them clearer.

- 5: Doesn't left panel have CI or are they too small?

The 95% CI intervals are too small to be visualised on the left panel of Figure 5. I have made this clearer in the figure legend.

**Reviewer #3**

Barnby and colleagues report their efforts to model attributions of intent arising during a series of dictator games, across the spectrum of paranoia in healthy people. They claim that the model recapitulates their behavioral results and that their analyses favor a specifically social interpretation of paranoia.

I am afraid I think this is a stretch and I feel more unpacking - and possibly further experiments - are necessary before sharing such a conclusion.

1) There are social/non-social behavioral manipulations in which people with higher paranoia have been shown to have more inflexible social beliefs without the need for modeling - eg Wellstein et al Schiz. Res. 2019. How do the present results compare to that work? It seems inappropriate to call the selfish attribusion non-social in the context of the current experiment and modeling. Can the authors unpack their reasoning here?

We agree with Review 3 that self-interest attributions are not non-social. We highlight this is in the text, specifically by stating that they are non-threat related attributions, which do not correlate with harmful intent attributions in the ‘true’ data both online and in the lab (Barnby et al., 2020a; Barnby et al., 2020b). We have reviewed the text to make sure this is clear throughout the manuscript, and first define it in the introduction:

“The true motivation for the decision is ambiguous but receivers rate the extent to which the decision has been motivated by harmful intent (a threat-related attribution about the partner) and the extent to which it is motivated by self-interest (a non-threat-related attribution about the partner) for each economic exchange. In this study, all receivers are paired with fair, unfair, and partially fair dictators for six rounds each in a counter-balanced fashion.”

In the discussion, we then compare our work with prior work that uses non-social behaviour.

While Wellstein et al., (2019) use a trust task to infer changes in those with differing baseline paranoia in a case-control-like study, we use a task that requires distinct inferences about a partner across the taxometric paranoia spectrum rather than trust behaviours. In addition, our computational modelling allows further empirical insight into individual changes of uncertainty and learning over time, rather than looking at group-wise effects as with Wellstein et al., (2019) – we mention this in both the introduction and discussion (e.g. line 347-349). We feel the fact that Wellstein et al., (2019) found converging results on a different task and a different design with a smaller sample without modelling adds strength to our conclusions.

2) 18 trials (comprised of six trials from each of three dictators) is extremely low. Can the authors show that their parameter estimates are stable and reliable?

The reviewer is absolutely correct. The parameters are not stable and reliable at the individual level, but they are at the population level. That is, a single individual cannot be placed on the basis of 18 trials. On the other hand, reliability forms a bound on validity, in our case external validity. In this population study the unreliability of the individual only matters to the degree that it obscures patterns over the population. In other words had we not been able to recover patterns over the population, the task would not have been reliable enough, but we can do so at high statistical significance, which demonstrates that for this purpose (as opposed to explaining a large proportion of the variance, or of locating individuals) the parameter estimates are reliable.

From previous guidelines about the reliability of computational models, we have established that the model is valid and reliable to infer latent statistics (Palminteri et al., 2017).

3) The table describing the meanings of parameter estimates is a welcome inclusion, unfortunately it is rather poorly written. It does emphasize that there are quite a few parameters in the models (almost as many as there are conditions). What are the effects on estimates/reproduction of behavioral features if some of those key parameters are removed?

The reviewer is right to wonder whether some of these parameters are redundant, i.e. if they could be condensed into one. However we believe that it is important to include a full spectrum of parameters, to allow the model to inform us about variability in the population with regards to (a) Harm Intent beliefs (b) Self-Interest beliefs and (c) the correspondence between belief and behaviour. Furthermore, we allowed each of these degrees of freedom to be characterized by magnitude and uncertainty, not only because these are of great psychological interest, but because in the Bayesian framework that we use the impact of observations (the dictator’s returns) on beliefs is only proportional to their uncertainty. Given our aims, we therefore had to include the four parameters pHI0, uHI0, pSI0 and uSI0. To minimize the number of parameters, we summarized all individual variation in one parameter with respect to the correspondence between belief and action, namely the expected variability or uncertainty of the latter, uΠ. This of course can cover a multiplicity of models of other’s strategy on behalf of participants, e.g. erratic, initially playing nicer than one is, etc., whose details were of no interest here.

The final parameter, the learning rate from one dictator to the next, was included for purely pragmatic reasons. Descriptive statistics showed that there was a powerful effect of Dictator order, and indeed if this parameter were omitted model fit deteriorated in the small pilot subsample (25 participants) that we used for model development.

Removing parameters would therefore have different effects at different levels of modelling:

- At the individual level, it would improve fit for those participant where the parameter obtains a ‘neutral’ value. An example is participants with near-zero fitted learning rates. There may also be subsets of participants where two parameters may be condensed: for example, the substantial correlations in fig. 4 may mean that the respective parameters might be condensed into one for subgroups of participants. This is a matter for further research.
- However, in the population such reductions in the number of parameters would hide the relationship between the psychological constructs concerned. For example, if uΠ were fixed to 1, one would not be able to detect its variation with GPTS. Note that each parameters furnished at least one significant relationship with GPTS, demographic variables or with each other.

Overall, it is highly likely many participants may be more parsimoniously described by a smaller set of parameters, but our results clearly show that each parameter used here is useful in describing some aspect of (co) variation of symptoms and behaviour in the population.

In addition, we have clarified the language in Table 1 to make it more readable and understandable.

4) The control analysis for the Network exploration is also a very good idea. Unfortunately, the authors seem to show that their fitting procedure is generating relationships between parameters. I suggest on that basis that the network analysis results be removed.

The simulation that we conduct to make sure the network analysis is not producing spurious results shows that the network fitting procedures do not produce correlations in the same direction as our ‘true’ data. Most relationships found are opposite or null and have very wide confidence intervals. Therefore, the real network analysis (titled ‘real network’ in the panel of Appendix D) can be shown to be detecting ‘real’ edges produced by examining pairwise effects controlled for by all the other relationships in the network model, and thus isn’t a product of spurious relationships generated in the model fitting process.

To add further assurances, and address the comments from the editor, we have included networks with fewer parameters in the supplementary materials to ensure the complexity of the network itself was not confounding our relationships of interest noted in our conclusions. We have included these to demonstrate that, in the absence of additional parameters that might confound the result, the relationships in the network moderated by GPTS score that are potentially less reliable (pHI0 -> eta, pHI0 -> upi) are consistent when removing parameters. We have referenced this in the main text (line 293-301):

“In addition, it may be that the complexity of the network itself is generating moderated effects between parameters due to redundant inclusion of variables around the relationships of interest (Stramaglia et al., 2014). To control for this possibility, we ran the Moderated Network Model with two, three, four, and five variables in the network to check whether the relationships between learning rate (eta), partner policy uncertainty (upi), and probability of attributing harmful intent (pHI0) still existed in absence of other relationships (see Supplementary Materials). We found that the variables in question were retained and were moderated in the same direction by GPTS score in all network models.”

5) More clarity on the behavioral features from Barmby et al (2020) that they are seeking to recapitulate with the model would be useful. What behavioral features should the model produce? How exactly does it produce them?

We have made it clearer in section 2.2 that the results listed are the statistical results found from previous analyses using the real data (i.e Barnby et al., 2020), and that this is what the simulated data should be compared to. We have recapitulated core behavioural features of the real data using simulated participants of the same size as our prior paper (n = 1794) – specifically, an effect of paranoia on harmful intent attributions within dictator conditions, and an effect of dictator conditions for both harmful intent attributions and self-interest attributions without interaction. This can be found described in section 2.2:

“A full analysis for the real data can be found in a previous paper (Barnby et al. 2020). In sum, Barnby et al., (2020) found that pre-existing paranoia increases harmful intent attributions (0.35, 95%CI: 0.15, 0.54), as does increasingly unfair dictator behaviour (2.00, 95%CI: 1.82, 2.18), and initially unfair dictator exposure leads to lower overall harmful intent attributions (-1.17, 95%CI: -1.52, -0.83). For self-interest, only increasingly unfair dictator behaviour (4.59, 95%CI: 4.26, 4.93), and initial dictator exposure (-0.71, 95%CI: -1.02, -0.39). These results should be the baseline comparison to assess our model’s ability to generate simulated data in section 2.3.”

6) Order effects of the dictators? Each human participant saw a fair, partially fair, and an unfair dictator in a random order. However, the model cares about these transitions, and, one would imagine, so do real participants. How well does the model capture the generalization from one dictator to the next? Does interacting with an unfair dictator first particularly impact highly paranoid people? Can the model capture that? It would seem that such an effect would be key to the authors interpretation of their prior published work, and it would be critical therefore that a learning model should capture this (such transitions from fair to unfair, for example, might bring these data in line with other approaches - like those of Nour, Wellstein, Reed - wherein volatility and participants responses to it seems to be key to paranoia - without such an analysis, I am not sure the present analysis has much to say about that other work and neither favorable nor unfavorable comparisons to that work are appropriate.

We agree with the reviewer that the order of dictators is crucial to our model, and indeed to participant inference and behaviour. Our prior work (Barnby et al., 2020) suggested that being presented with an increasingly unfair dictator first (unfair > partially fair > fair) led to reduced harmful intent attributions over all in the game.

Our model, detailed in the methods, and described in an earlier response to your comment (3), takes into account the order of dictators’ participants saw, and indeed the order of trials participants saw in the partially fair condition. Without this, the model isn’t able to capture the data to the same degree.

The models parameter ‘learning rate’, as described in Table 1, is an empirical metric of how similar dictators were believed to be in terms of their intentions:

“A higher **η** leads the starting assumptions of dictators after the first one seen to be influenced by the evidence seen. It can be thought of as a strength of belief that the Dictators seen during the experiment will resemble each other.”

7) Parameter recovery. The simulated data produce simulated behavioral ratings. If the authors fit their model back to those simulated data what is the relationship between the estimates from real behavioral data and simulated data?

As highlighted in an earlier response, our model is able to capture reliable parameters at the population level.

In addition, in section 2.3 we fit the same regression models to the simulated data as we did for our real data (listed in 2.2). We find that the simulated data is able to recapitulate the same effects as the real data.

Finally, we have created correlations between attributions for simulated participants and real participants for each division of GPTS score and added this to the supplementary materials (Appendix H). All correlations are significant and at least at rho = 0.83 for harmful intent attributions, and rho = 0.64 for self-interest attributions. We have included this in the text:

“Simulated attributions were also highly correlated (harmful intent, rho = 0.83 – 0.9; self-interest, rho = 0.64 – 0.7, ps < 0.0001) with real participant attributions for all levels of GPTS score (as defined in Barnby et al., 2020).”

**Reviewer #4**

I would like to thank the authors for an exciting read! The paper was well structured and the methods were interesting and appropriate for the data they were used on. The points I am raising mainly concern clarity on a few details and open questions.

1. *Introduction and Methods:*

Could you provide the reader with the instructions the participants received regarding the game and the dictators? This is not only important for replication of these results but also in order to understand better, what the participants might have based their inferences on.

We will include in the supplementary materials a full list of the instruction’s participants received as well as a brief task schematic to clarify this point.

Did participants know that they would be playing against different types of dictators?

Participants were informed that they would be playing against three different players for 6 rounds each. This was clarified in the instructions listed in the supplementary materials.

Do you have any demographic data that you can provide for this sample?

All of the demographic data of these samples are provided in the prior publication that used this data (Barnby et al., 2020).

Why did you only use the two types of attributions you mentioned? Couldn’t some participants have assumed that there were no real intentions behind the dictator behavior but that it was just how the game was set up?

Prior work that has used shorted versions of this modified Dictator game has found the same robust behavioural effects (Raihani & Bell, 2017a; 2017b) as we did in our prior publication (Barnby et al., 2020), and methodologically we wanted to use the same attributions as previous work as a validity check. Conceptually, we also included these attributions so that participants were able to make differential potential inferences about their partners behaviour that could be argued – they behaved the way they did because they were self-interested, but also possibly because they wanted to harm the participant. We might argue that if participants felt there were no real intentions by their partner, they would have scored both attributions as 0 (not at all self-interested, not at all intending to harm the player).

You treat HI and SI as independent could you elaborate on why you assumed that these two types of attributions are independent? After all they did correlate with each-other and also conceptually they do seem to share common ground.

Both harmful intent and self-interest are not correlated in the real data in this experiment, nor are in prior studies (Barnby et al., 2020a; 2020b) that used this task in an online and lab setting, and further only the simulated data in this experiment was correlated to a very small degree (rho = -0.06; 0.036% of the variance). In addition, we only see a reliable correlation over multiple tasks of pre-existing paranoia with harmful intent but not self-interest (Raihani & Bell, 2017a; 2017b; Barnby et al., 2020a; 2020b), and therefore have good reason to believe that they are independently made inferences.

*Introduction, Lines 38-43: “More recently, Barnby et al. (2020) extended this work and additionally found that individuals high in paranoia were more likely to specifically reduce high harmful intent attributions after an initial peak when interacting with partners who were consistently fair, when compared to people low in paranoia, potentially suggesting increased volatility of paranoid social inferences.”*

I have read this sentence three times now but still cannot follow the reasoning. Maybe it’s too long of a sentence. Would you mind making it easier to follow what you found and how this relates to your hypothesis regarding volatility?

We have adjusted this paragraph to be clearer and to relate it to our hypotheses.

*Introduction, Lines 89-90:* “*Second, we tested whether baseline harmful intent attributions about the partner depended on pre-existing paranoia.”*
What do you mean by baseline? The very first attribution?

We have removed ‘baseline’ – this was an error – thanks for noticing it.

*Table 1, uHI0:* “*Greater uHI0 denotes weaker assumptions harmful intent, and hence greater willingness to believe that a Dictator less generous than expected has higher intent to harm.”* Shouldn’t this be the other way around? Intuitively, a larger spread of the distribution should be linked to greater uncertainty regarding the harmful intent attribution, which should result in a smaller likelihood of this attribution being made?

The statement we have made about uHI0 is correct – larger values denote larger individual distributions of harmful intent, and therefore that, overall, a participant is less certain about a dictator’s harmful intent given their behaviour.

*Table 1, uΠ (Partner policy uncertainty):*
Here you are not describing what type of quantity this is. Uncertainty has been analogous to the spread of a distribution before, which is not the case here. For clarity and consistency, it might be beneficial to specify this here.

We have adjusted this description in the text for clarity.

***Table 1, η: “****A higher η leads the starting assumptions of dictators after the first one seen to be influenced by the evidence seen. It can be thought of as a strength of belief that the Dictators seen during the experiment will resemble each other.”*

These two sentences sounded contradictory to me at first and second glance. Maybe because you don’t state what you mean by “evidence”. My first interpretation was that you mean trial- by-trial information but you might mean something different?

We have adjusted this description in the text for clarity. We want to be clear that learning refers to the assumptions made by a participant at the start of interaction with a new dictator, given the inferences made so far about dictators they’ve seen before, and therefore how much prior dictator behaviour influence current inference assumptions.

***Results, general:***

Would you mind stating effect-sized for the results you are reporting?

We have now included effect sizes for all the results that are different from the beta coefficient.

I know from your previous paper that you divided participants into “low”, ”medium”, ”high”, “very high”, “clinical” regarding GPTS scores. However, this is not clear at all when only reading this paper. Could you mention that somewhere, i.e. what the score cut-offs for each of these groups were and why you chose these particular cut-offs and bins?

In this particular paper we did not use cut offs from GPTS scores to calculate effects in our parameter associations and global statistical models. Instead we included GPTS as a scaled and centred continuous variable. We have made this clearer in the methods (e.g. 579-580). We only use GPTS cut offs for the purposes of visualisation in simulating the behaviour data in Figure 2.

How many participants attributed HI and SI equally often or seemed to choose their attributions randomly? It might be possible that some participants might have believed that this was all computer-generated and thus did not really try to answer these attribution questions. Would that be reflected in the policies and if so, could you provide a plot for the mean partner policies over trials?

A log likelihood of -4.394 in our model indicated that scores were attributed randomly and not in response to partner policies. At no point did the entire model, individual trials, and GPTS scores significantly drop below a log likelihood value of -4.394, therefore we are confident that the majority of the sample answered non-randomly.

Likewise, we found in the behavioural data that harmful intent attributions were adjusted preferentially with paranoia, and both harmful intent attributions and self-interest attributions were significantly affected by dictator behaviour. If participant inference was random and not informed by partner policy, we’d expect no such relationship between attributions and dictator behaviour, or pre-existing paranoia.

***Results, Lines 111-116:***

*“Linear mixed model analyses using ID...”*

could you spell out “ID” the first time you use it in the text body? I assume you mean the participant identification number, correct?

We have clarified this in the text.

*“...as a random term suggest that as trials progressed overall from one to eighteen (-0.012, 95%CI: -0.021, -0.003) and at higher values of the GPTS (-0.005, 95%CI: -0.007, -0.003) participants were less able to be predicted by our model, ...”*

Is this based on a regression on the GPTS scores or based on divisions of the GPTS scores? If the latter is the case, how did it enter the mixed model?

GPTS was entered into all models as a scaled and centred Z score – we have made this clearer in the text.

Across all types of dictators?

These models take into account all dictators as trials 1-18 include the three different types of dictators implicitly within them.

*“...whereas our model was better able to predict behaviour from partially fair (0.43, 95%CI: 0.32, 0.55) and unfair (0.31, 95%CI: 0.25, 0.55) dictators than fair dictators.”*

Better than what? And in this case now across all GPTS scores?

The unfair and partially fair dictators are included as an ordered factor in the mixed models, and therefore are held up against the fair dictator as comparisons. This statement is suggesting that compared to fair dictators, the model was able to capture the other two dictators to a higher degree.

***Figure 1, general:***

It would increase clarity greatly if you could add titles to each subfigure and in the legend mention that the horizontal line refers to chance-level.

We have added titles to each subfigure and included in the legend that the grey line represents chance level of likelihood.

***Figure 1, C and D:***

I assume that the Scale (Low to Clinical) relates to the GPTS scores? Is that the way you divided the groups then? This does not immediately become clear when looking at this figure. In the legend you refer to plotting the mean log likelihood for each dictator, I cannot see this here and also cannot see anything with reference to this scale.

I will make it clear in the figure legend that the GPTS score cut offs are only for visualisation purposes. Likewise, the figure colours the top two panels (A&B) but doesn’t give scores for each dictator overall. We have made this clearer in the legend.

***Results, , Lines 138-144:***

*“Simulated (n = 1754) harmful intent attributions and self-interest attributions were only slightly negatively correlated with each other overall (rho = -0.06, p < 0.001).”*

This implies that you expected and were aiming for this negative correlation. Is that so and why?

We have previously been questioned in peer-review over whether attributions are truly independent of one another and to demonstrate their independence with a correlation. We wanted to add this correlation for transparency and to show that attributions only show a slight relationship in the simulated data, and in previous data show no correlation with each other (Barnby et al., 2020a; Barnby et al., 2020b).

*“Pre-existing paranoia in all dictator conditions increased harmful intent attributions (Unfair: 0.21, 95%CI: 0.18, 0.25; Partially Fair: 0.19, 95%CI: 0.16, 0.23; Fair: 0.18, 95%CI: 0.15, 0.22). “*

Paranoia increasing harmful intent attributions implies that they were lower at some point before where there was no paranoia, because the word is used as a verb. It’s also not clear in reference to what the attributions were increased, are you comparing the clinical GPTS scores to all others or is this across all scores? Does this result refer to all or only a specific dictator?

We are including the pre-existing paranoia as a continuous z-score in all of the models (which has now been made clearer in the text), and therefore this refers to how harmful intent attributions changed as pre-existing paranoia increased along the continuum for each dictator condition. From other analyses in the paper that we’ve conducted and from our previous data (Barnby et al., 2020) this is also shown to be true across the entire task. We will this clearer in the text, e.g. line 166.

*“Pre-existing paranoia was only a predictor of slightly reduced self-interest attributions in unfair dictators only (-0.08, 95%CI: -0.12, -0.04), using individual cumulative link models with age and first dictator exposure as additional predictors.”*

I apologize for my ignorance, but I could not find out what individual cumulative link models are, would you mind explaining that in one or two sentences or provide a reference?

Cumulative link models (and cumulative link mixed models) are equivalent to general linear models or general linear mixed models, however, allow for ordinal or factored variables to be used as a dependent variable in the regression equation (see Christensen, R. H. B. (2015). ordinal—regression models for ordinal data. *R package version*, *28*, 2015. for the theory and statistical R package used.)

***Results, Lines 437-440* and *Lines 440-448:***

*“The prior belief over both HI and SI can then be written as a product of the independent prior probabilities, p(0)HI * p(0)SI . This assumption of independence is conservative, minimizing the number of free parameters.”*.
“*We then assumed that HI=0, SI=0 resulted would correspond to a preference in giving r=n (of n, i.e. 100%) to the participant ('self-sacrifice'), HI=1, SI=1 to a high preference for giving r=1 (of n, i.e. 0%) to the participant, but very high SI or HI with moderate scores on the other dimension we sufficient for a substantial probability of r=1 of n (i.e. 0%).”.*

Maybe I am misunderstanding something here, but first you state that you treat HI and SI as independent but in the next section you state that their mapping from dictator behaviour to beliefs are the same. Are you not then saying that they actually are NOT in fact independent?

We apologise for the lack of clarity here. There are two issues:

a. The former, *p(0)HI * p(0)SI*, are prior beliefs or assumptions that participants hold about others, not HI and SI formed on the basis of dictator behaviour. It is perfectly possible in our model and in real life for a participant to hold uncorrelated baseline assumptions about other’s intentions but for correlations to emerge on the basis of observations.
b. What is described in the paragraph “We then assumed that *HI=0* …” is not a map *from behaviour to beliefs*, but the other way around, *from attributes to behaviour*. When it comes to belief updating, the distinction is very important: the model that a participant hold about dictator-attribute-to-behaviour *has to be inverted by the participant* upon observing returns in order to update beliefs about attributes. Again, it is perfectly possible in our models (as in real life) for a behaviour (say, an unexpected generous return) to asymmetrically change beliefs about selfishness (‘hmm, this person is likely to be less selfish than I thought) or about harm-intent (‘likely less nasty than I thought’). In our models, the degree of symmetry will depend on the relative certainty about attributes.

‘In order to make inferences based on the feedback they get from dictators, participants must also hold a correspondence between attributes and behaviours. We emphasise that participants hold maps *from attributes to behaviour*, and not directly from observations of returns to attributes. Thus, they have invert these maps to update their beliefs, which will typically result in asymmetric belief update depending on further detail (so that Eq. uses full joint probabilities, breaking the initial independence *p(0)(HI,SI) = p(0)HI * p(0)SI*). To build a map from attributes to behaviour that could capture a full range of possibilities, and thus could be used to describe all participants, we first provided for a range of possible dictator behaviours, discretizing returns using a similar resolution as attitudes. *HI*=0, *SI*=0 corresponded to the attributions of each participant which would result in a dictator preference to give *r (return)*=*n* (of *n,* i.e. 100%) to the participant ('self-sacrifice'). At the top end of the scale, *HI*=1, *SI*=1 were attributions resulting in a high preference for giving *r*=1 (of *n*, i.e. 0%) to the participant. Very high *SI* or *HI* with moderate scores on the other dimension we sufficient for a substantial probability of *r*=1 of n (i.e. 0%).’

I assume “r” stands for reward. This is the first time this comes up in the text body, please indicate its meaning when you are using it for the first time.

‘r’ stands for ‘return’ – we have clarified this in the text when it first appears.

***Results, Lines 458-460:*** “*This means that every modelled participant had the same basic repertoire of attribute behaviour available to them, and that HI and SI can be seen as ideographically scaled.”*
Apologies for my ignorance again. What does ideologically scaled mean?

‘Ideographically scaled’ means that each attribute (HI, SI) can be represented on a scale for each participant individually. We will make this clearer in the text.

***Results, Lines 496-498:*** *“In contrast to a model selection approach, the ability of a model to simulate data is necessary to assess its validity and falsification (Palminteri et al., 2017).”* Why is that ‘in contrast to model selection?’ Are they competing approaches?

They are not directly comparing model selection approaches and model generative ability, as may be understood in our text. We were trying to say that while we do not employ model selection approaches, our methods of assessing model validity is itself a reasonable approach. We have cleared this up in the text to avoid ambiguity.

***Results, Lines 279-282:*** *“Our findings converge with the idea that paranoia strengthens higher-level beliefs about others to influence momentary inferences regardless of a partner’s behaviour (Wellstein et al., 2019).”*
Based on what results do you reach this conclusion? It is not entirely clear to me.

In both the Wellstein (2019) and our paper, we find that pre-existing paranoid beliefs (higher order beliefs about others) influence momentary inferences in our live task. We have cleared this up in the text to avoid ambiguity.
